# Prior cancer history and suitability for kidney transplantation

**DOI:** 10.1093/ckj/sfad141

**Published:** 2023-06-28

**Authors:** Germaine Wong, Wai H Lim

**Affiliations:** Sydney School of Public Health, University of Sydney, Camperdown, NSW, Australia; Centre for Kidney Research, Kids Research Institute, The Children's Hospital at Westmead, NSW, Sydney, Australia; Centre for Kidney and Transplantation Research, Westmead Hospital, NSW, Sydney, Australia; Department of Renal and Transplantation Medicine, Sir Charles Gairdner Hospital, WA, Perth, Australia

**Keywords:** cancer, recurrence, shared decision-making, survival, transplantation

## Abstract

Kidney transplantation is the optimal treatment for most patients with kidney failure. For patients with a prior history of treated cancers, listing and transplant eligibility decisions are complex. Patients and health professionals are obliged to consider the time-periods between cancer cure and transplantation, the risk of cancer recurrence under the influence of immunosuppression and anti-cancer treatment options if the disease recurs. Cancer recurrence is associated with a high mortality rate, thus potentially reduces the projected survival benefit of transplantation, and dampens the utility of scarce organs. In view of the uncertain risk of harms, clinicians may consider transplantation for candidates with prior cancer history only after an extended period of cancer-free interval, as the fear of disease recurrence and shortened life expectancy may outweigh the benefits of receiving a kidney transplant compared with dialysis. Over the past decade, the evolution of novel anti-cancer therapies coupled with improved understanding of cancer genomics have led to considerable improvement in cancer-free survival. It is therefore justifiable to make individualized transplant suitability decisions based the joint effects of cancer biology, available therapeutic options and prognostic covariates on clinical outcomes. In this review, we first summarized the cancer epidemiology in kidney transplant recipients. We then explored how the probability of cancer cure, risk of recurrence and outcomes in candidates with a prior cancer history may influence the decisions to transplant. Finally, the role of shared decision-making between health professionals and patients regarding the optimal management options, and considerations of patients’ preferences and values are discussed.

## EPIDEMIOLOGY OF POST-TRANSPLANT MALIGNANCY IN KIDNEY TRANSPLANT RECIPIENTS

Cancer is a devastating complication for patients with kidney transplants. Cancer is one of the most important outcomes to be included in clinical trials by key stakeholders including patients, caregivers, health professionals and policymakers [1]. This is because the overall cancer incidence is increased by approximately 2–4 times compared with the age- and sex-matched general population [2, 3]. More importantly, the increased risk is site specific, with the greatest risks incurring for immune-driven and viral related cancers such as Kaposi sarcomas, anogenital cancers and post-transplant lymphoproliferative cancers (PTLD). The excess risk compared with the general population for Kaposi sarcomas and haematological malignancies such as lymphoma exceeds 50 and 10 times, respectively, compared with the age- and sex-matched general population. The cumulative incidences of any cancer types vary from 5% during the first year post-transplant, to 15% after 10 years and >50% 25 years after transplantation [4]. Apart from the increased incidence, survival after cancer diagnosis is also significantly worse in transplant recipients than those without kidney transplants. The overall standardized mortality ratio is approximately 2–3 times higher than that of the general population, and the relative risk of death is highest among younger transplant recipients [5]. Reports from registry analyses indicated that the relative risk of cancer-related death among young recipients aged 20–29 years is 18 times higher than the age-matched general population but a declining trend in the overall relative increased risk of cancer death is observed with increasing recipient age. Older transplant recipients (aged 65 years and above) experienced approximately a 2-fold increased risk of death compared with similar age groups in the general population [6]. Recent data from registry studies showed that the elevated risk of cancer and cancer-related deaths was further exacerbated with repeated transplants and allograft losses. In a registry-based cohort study of dialysis and transplant patients comprising 274 660 total person-years of follow-up, the overall risk of cancer and cancer mortality doubled during periods with a functioning first or second kidney transplant, compared with the age- and sex-matched general population [7]. After the loss of the first kidney transplant, there was a reduction in the relative cancer incidence and mortality risk compared with recipients with a functioning first kidney transplant, but these estimates remained higher than that for the general population. On the contrary, the overall cancer incidence and cancer-related death risk ratio remained elevated after the loss of the second allograft, with an excess risk of at least 2.5 and 3.5 times for cancer incidence and cancer-related death compared with the general population [7].

There are many reasons for the observed poorer outcomes in transplant recipients compared with the general population. Some have suggested that cancers in transplant recipients are less responsive to treatment, particularly with the use of immunotherapy as the immunosuppression used to maintain allograft function and survival may diminish the efficacy of immunotherapy. In addition, enhancement of the immune system caused by the checkpoint inhibitors could lead to allograft function [8]. The reported incidence for acute rejection with the use of anti-programmed death ligand (PD-L1) and anticytotoxic T-lymphocyte-associated protein 4 (CTLA-4) drugs varies between 50% and 60% in kidney transplant recipients [9]. Other potential factors that may be associated with poor cancer prognosis include the presence of co-existing comorbidities, reduced kidney function, late presentation due to the lower uptake of preventive strategies and screening [10, 11], and aggressiveness of the malignancy under influence of immunosuppression. Kidney dysfunction is common in patients with cancer and kidney transplants. Most of these patients have mild to moderate stage chronic kidney disease. In clinical practice, dose adjustments in accordance with kidney function are required to prevent drug toxicity [12]. However, establishment of the appropriate estimation of kidney function has been challenging. The recently published international consensus guideline for anticancer drug dosing recommends the use of estimated glomerular filtration rate (eGFR) calculated via the Chronic Kidney Disease Epidemiology Collaboration (eGFR_CKD-EPI_) equation to guide the assessment of kidney function in adult cancer patients, except where directly measured glomerular filtration rate (mGFR) is clinically necessary [13]. Directly measured GFR, expressed in mL/min, refers to a direct measurement of the clearance of exogenous markers (filtered by the glomerulus and neither reabsorbed nor secreted by the kidney tubules), such as iohexol, iothalamate, 51Cr-EDTA or 99mTc-DTPA. While mGFR is probably the most accurate method for kidney function assessment for chemotherapeutic drugs dosing, it is not always practical and cost-effective to implement in all clinical circumstances [14]. Therefore, mGFR is reserved for situations such as obesity, sarcopenia, paraplegia or amputees, where calculating eGFR_CKD-EPI_ may be unreliable [14]. Underestimation of kidney dysfunction may result in unnecessary dose reductions or a switch to an alternative agent which are less effective and may influence the overall patient survivals [12].

## STRATEGIES TO PREVENT CANCERS AFTER KIDNEY TRANSPLANTATION

In view of the poor prognosis of kidney transplant recipients with cancer, preventive strategies such as vaccinations [the human papillomavirus (HPV) vaccines for HPV infection–related anogenital cancers], routine pre- and post-transplant cancer screening, as well as careful assessments of all potential living and deceased kidney donors to prevent inadvertent transmission of donor-derived diseases such as cancer are in place to reduce the risk of *de novo* cancer occurrence in transplant recipients. Among recipients with a prior cancer history, recommendations by most clinical practice guidelines suggested a waiting time of 2–5 years from the time of cure (complete remission) to transplantation, depending on the cancer site and the initial stage of disease [15]. However, there is substantial variability in these recommendations between countries and regions [6]. For example, in Norway, the waiting time prior to listing for most cancer is 1 year from the time of complete remission. On the contrary, the recent Kidney Disease: Improving Global Outcomes (KDGIO) guideline suggested a minimal wait-time of at least 2 years from cure, except for indolent and low-grade cancer, such as those with cancer prostate cancer (Gleason score ≤6), superficial nonmelanoma skin cancer and incidentally detected renal tumours (≤1 cm in maximum diameter) [16].

Table 1 shows the reported rates of cancer incidence and deaths in the general populations and among kidney transplant recipients with prior cancer history, focusing on the more common cancer types listed in the updated 2020 KDIGO guidelines for potential kidney transplant candidates [16]. The revised recommendations for the transplant eligibility of patients with prior cancers were underpinned by level two or ungraded evidence (opinions of a selected consensus group which may or may not have direct experience in real world practice). Therefore, this evidence is subjected to confounding, selection and indication biases. There are three key observations from these guidelines: (i) pre-transplant population-specific cancer screening is recommended, but the clinical utility of other cancer screening processes outside routine practice and recommendation is unknown; (ii) the recommended waiting time between cancer remission and transplantation is based on a categorization of major cancer subtypes, without consideration of cancer genomics, biology and treatment characteristics of prior cancer or rarer cancers; and (iii) patients with a history of ‘cured’ metastatic cancer should not be excluded for transplantation, but transplant professionals and patients should balance the risk of cancer recurrence, risk of cancer death if disease recur, the risk of death on dialysis from other causes against the projected survival benefits and quality of life gains associated with kidney transplantation.

**Table 1:** Risk of cancer recurrence and survival in the general population and kidney transplant recipients.

	KDIGO 2020 guidelines		General population		Kidney transplant recipients	
Cancer type	Stage	Wait-time interval (years)	Incidence/mortality (global ASR^a^)	5-year relative survival	Cancer recurrence	Recommendation
Breast	Early	≥2	47.8/13.6 (female)	Localized: 99%	2.0 per 100 person-years (95% CI 1.3–3.3) [21]^c^	Localized ≤2 years
	Advanced	≥5		Regional: 86%		Regional ≤5 years
				Distant: 30%		Distant >5 years
Colorectal	Dukes’ A/B	≥2	19.5/9.0^b^	Localized: 91%	4.7 per 100 person-years (95% CI 1.7–12.4)—include all GI tumours [21]^c^	Localized ≤2 years
	Duke C	2–5		Regional: 73%		Regional ≤5 years
	Duke D	≥5		Distant: 15%		Distant >5 years
Bladder	Invasive	≥2	5.6/1.9	*In situ*: 96%Localized: 70%Regional: 39%Distant: 8%	2.2 per 100 person-years (95% CI 0.2–27.0) [21]^c^	*In situ*/localized ≤2 yearsRegional ≤5 yearsDistant >5 years
Kidney	<3 cm	0	4.6/1.8	Localized: 93%	2.2 per 100 person-years (95% CI 0.8–6.2) [21]^c^	Localized ≤2 years
	Early	≥2		Regional: 72%		Regional ≤5 years
	Large/invasive	≥5		Distant: 15% (includes renal pelvis)		Distant >5 years
Uterine	Localized	≥2	8.7/1.8 (female)	Localized: 95%	0.4 per 100 person-years (95% CI 0.0–150.0) [21]^c^	Localized ≤2 years
	Invasive	≥5		Regional: 70%		Regional ≤5 years
				Distant: 18%		Distant >5 years
Cervical	Localized	≥2	13.3/7.3 (female)	Localized: 92%	3.9 per 100 person-years (95% CI 1.6–9.3) [21]^c^	Localized ≤2 years
	Invasive	≥5		Regional: 59%		Regional ≤5 years
				Distant: 17%		Distant >5 years
Lung	LocalizedRegional/distant	2–5	22.4/18.0	Localized: 61%Regional: 34%Distant: 7%	5.4 per 100 person-years (95% CI 1.7–16.6) [21]^c^	Localized/regional >5 yearsDistant—contraindicated
Testicular	Localized	≥2	1.8/0.2 (male)	95%	0.7 per 100 person-years (95% CI 0.2–2.3) [21]^c^	Localized ≤2 years
	Invasive	2–5				Regional ≤5 years
Melanoma	Localized	≥5	3.4/0.6	Localized: 99%	1.9 per 100 person-years (95% CI 0.8–4.7) [21]^c^	Localized ≤2 years
	Invasive	Contraindicated		Regional: 71%		Regional ≤5 years
				Distant: 32%		Distant >5 years
Prostate	Gleason ≤6Gleason 7Gleason 8–10	0≥2≥5	30.7/7.7 (male)	Localized: 100%Regional: 100%Distant: 32%	0.8 per 100 person-years (95% CI 0.1–12.5) [21]^c^	Localized/regional ≤2 yearsDistant ≥5 years
Thyroid	Stage IStage IIStage IIIStage IV	0≥2≥5Contraindicated	6.6/0.4	Localized: 100%Regional: 98%Distant: 53%	1.8 per 100 person-years (95% CI 0.2–12.8) [21]^c^	Localized/regional ≤2 yearsDistant >5 years
Hodgkin lymphoma	Localized	≥2	1.0/0.3	Stage I: 92%	9% [35, 36]	Stage I/II ≤2 years
	Regional	3–5		Stage II: 95%	1.3 per 100 person-years (95% CI 0.2–10.0) [21]^c,d^	Stage III/IV ≤5 years
	Distant	≥5		Stage III: 86%		
				Stage IV: 80%		
Non-Hodgkin lymphoma	Localized	≥2	5.8/2.6	Stage I: 87%	11% [35, 36]	Stage I
	Regional	3–5		Stage II: 78%	1.3 per 100 person-years (95% CI 0.2–10.0) [21]^c,d^	Stage II/III ≤5 years
	Distant	≥5		Stage III: 72%		Stage IV >5 years
				Stage IV: 64%		
PTLD (in kidney transplant recipients)	NodalExtra nodal and cerebral	≥2≥5	Not reported	Overall survival (prior kidney transplant): 63% at 1 year, 55% at 5 years [37]	2.8% (7/254) after second kidney transplant (with history of PTLD) [38]	Nodal ≤5 yearsExtra-nodal >5 years
				5-year survival [37]: nodal–64%; bone marrow–23%; CNS–42%; allograft–62%; other extra-nodal–49%		
NMSC		No recommendation	Not reported	BCC: 100%, SCC: 95% [39]	Geographical variation: Italy (5% after 5 years and 10% after 10 years); Northern Europe (10% after 10 years and 40% after 20 years); Australia (45% after 11 years and 70% after 20 years) [41, 42]	Localized ≤2 years
				2-year survival [40]: locally invasive SCC <25%, metastatic <10%		Locally invasive SCC >5 years
						Metastatic SCC—contraindicated
Multiple myeloma		No recommendation	1.8/1.1	Localized: 79%	50% [43]	
				Distant: 57%		
Brain		No recommendation	3.5/2.8	Localized: 35%	No data	
				Regional: 21%		
				Distant: 30%		
Ovary		No recommendation	6.6/4.2 (female)	Localized: 93%	No data	Localized ≤2 years
				Regional: 74%		Regional ≤5 years
				Distant: 31%		Distant >5 years
Stomach		No recommendation	11.1/7.7	Localized: 72%	No data	Localized ≤5 years
				Regional: 33%		Regional >5 years
				Distant: 6%		Distant—contraindicated
Liver		No recommendation	9.5/8.7	Localized: 36%	No data	Localized >5 years
				Regional: 13%		Regional/distant—contraindicated
				Distant: 3% (includes intrahepatic bile duct)		
Oesophagus		No recommendation	6.3/5.6	Localized: 47%	No data	Localized >5 years
				Regional: 26%		Regional/distant—contraindicated
				Distant: 6%		
Lip/oral cavity		No recommendation	4.1/1.9	Lip: localized: 94%; regional: 63%; distant: 38%	No data	
				Tongue: localized: 84%; regional: 70%; distant: 41%		
				Oral cavity and pharynx: localized: 86%; regional: 69%; distant: 40%		
Leukaemia		No recommendation	5.4/3.3	66% (ALL: 71%, AML: 31%, CLL: 88%, CML: 70%)	No data	

^a^ASR (2020 data): age-standardized rate (both sexes).

^b^Includes anal cancer (https://gco.iarc.fr/today/home). SEER based on SEER 17 2012–18 data (https://seer.cancer.gov).

^c^Includes all solid organ transplant recipients.

^d^Estimates of all haematological cancers.

GI: gastrointestinal; NMSC: non-melanoma skin cancer; BCC: basal cell carcinoma; SCC: squamous cell carcinoma; ALL: acute lymphocytic leukaemia; AML: acute myeloid leukaemia; CLL: chronic lymphocytic leukaemia; CML: chronic myeloid leukaemia.

In 2021, the American Society of Transplantation published a consensus and conference workshop report that aimed to assist transplant professionals in the evaluation of solid organ transplant recipients with a prior cancer history including breast, colorectal, anal, urological, gynaecological and non–small cell lung cancers [17]. Given that this is a consensus meeting report, the evidence underpinning the recommendations was not graded and an overall GRADE (Grading of Recommendations, Assessment, Development, and Evaluations) quality rating was not applied to the body of evidence across all outcomes assessments. Like the KDIGO recommendations, this consensus expert opinion statement recommends a personalized approach to evaluating a potential transplant candidate with prior cancer history, suggesting consideration of all factors, including patient, organ specific, immunosuppression and cancer-specific factors, prior to listing. These may include the expected survival of recipients, the type of solid organ transplants they received and whether the transplants were lifesaving (such as heart and lung transplant) versus life-prolonging (such as kidney transplant), cancer stage at diagnoses, the genetic risks, patients’ responses to treatment and the risk of disease recurrence after remission. A 5-year cancer survival rate of near 80% was assumed to be an acceptable benchmark before proceeding with transplantation.

## PROBABILITY OF CANCER CURE IN PATIENTS WITH KIDNEY FAILURE AND CANCER

Accurate evaluation of the predicted cure probabilities of cancer at the time of transplantation in patients with a prior cancer history is crucial because it provides appropriate guidance and strategies to minimize the risk of cancer recurrence after transplantation. Recent data have shown that the cure probabilities of specific cancers pre-transplant may accurately predict the risk of cancer-related death after transplantation. In this registry analyses of over 10 000 000 patients, 17 cancer types were assessed. At the time of transplant, the mean cure probability among transplanted patients with cancer was 94% and was considerably higher than that in patients with cancer in the general population [18]. Transplant recipients with low cure probabilities were more likely to have a previous diagnosis of oral cavity or pharyngeal, lung, breast, bladder or kidney cancers, or multiple myeloma. In addition, these patients were also more likely to have advanced stage cancer, have had shorter intervals between cancer diagnosis and transplantation (median 3.6 years compared with 8.6 years) and to be older at cancer diagnosis (median age 57 years compared with 51 years). Moreover, these cure probabilities may correlate with post-transplant cancer-specific mortality. The risk of cancer-related death doubled among recipients with cancers of low cure probabilities compared with those with high cure probability, but risk of non-cancer related death was not associated with the cure probabilities of cancer [18]. For recipients with a prior history of haematological malignancy such as multiple myeloma, treatments with chemotherapy and immunomodulating agents such as lenalidomide, or other proteasome inhibitors and monoclonal antibodies, may control the progression of disease, but these therapies are not curative [19]. Prior recommendations have suggested patients with multiple myeloma and cast nephropathy in remission after treatment are not suitable for transplantation because of a higher risk of disease recurrence and poor outcomes once disease recurs. However, few case reports have reported success in achieving remission and subsequent kidney transplantation in patients with a history of multiple myeloma [20]. Among those with kidney failure and multiple myeloma, a potential strategy to minimize complications and maximize survival is to conduct a human leukocyte antigen (HLA)-matched, combined kidney and stem cell transplantation. Among candidates with multiple myeloma and without an HLA-identical living kidney donor, treatment with chemotherapy, follow by autologous stem cell transplant may be a viable option to achieve remission prior to kidney transplantation.

## PROBABILITY OF CANCER RECURRENCE IN KIDNEY TRANSPLANT RECIPIENTS WITH PRIOR CANCER HISTORIES

Applying and extrapolating the statistical cure estimates from the general population in the transplant population is not without limitations. Given the influence of immunosuppression, the probability of cancer recurrence after kidney transplantation in patients with prior cancer(s) remains uncertain, as the estimates of cancer recurrence were informed by historical data, and selection bias was unavoidable. More importantly, the influence of novel anti-cancer treatment options on survival was not considered. Despite the inherent limitations, these estimates supported the current recommendations and guidelines for transplant eligibility. In a systematic review of 57 cohort studies (with inclusion of studies until December 2016) comprised of kidney and non-kidney solid organ transplant recipients [19 (33%) studies of kidney transplant recipients and 17 (30%) studies of various solid organ transplant recipient], the proportion of recipients with pre-transplant cancer and had experienced cancer recurrence post-transplant ranged between 0.4% and 22%, with a pooled proportion of 5% (95% CI 3%–8%). In the corresponding meta-analysis of 22 studies, cancer recurrence rate was estimated to be between 0.3 and 15.4 events per 100 person-years at risk, with the pooled estimate of 1.6 events per 100 person-years (95% CI 1.0–2.6). A high degree of study heterogeneity was observed (I^2^ = 87%), and was explained by the year of publication, risk of bias and length of follow-up period. Subgroup analysis reported that kidney transplant recipients (1681 recipients with pre-transplant cancer, 2.4 events per 100 person-years, 95% CI 1.0–5.6; I^2^ = 95%) experienced the highest recurrence rate compared with recipients of non-kidney transplants, with the pooled risk ratio of 2.80 (95% CI 1.12–7.01; I^2^ = 65%) for cancer recurrence if the interval between cancer and transplantation was >5 years, as compared with an interval of <5 years [21]. Other population-based cohort studies showed that kidney transplant recipients with a history of pre-transplant skin and non-skin malignancies have a significantly higher risk of cancer recurrence and *de novo* cancers after transplantation, independent of the time interval between pre-transplant cancer occurrence and transplantation [22, 23].

## PROBABILITY OF CANCER-SPECIFIC AND ALL-CAUSE DEATH IN KIDNEY TRANSPLANT RECIPIENTS WITH PRIOR CANCER HISTORIES

Transplant recipients with a prior cancer history may experience a significant survival disadvantage compared with recipients without a cancer history [22–24]. In a meta-analysis of 23 cohort studies (with inclusion of studies until December 2015) of solid organ transplant recipients with pre-transplant cancer, the pooled hazard ratio (HR) for all-cause mortality in these recipients with pre-transplant cancer (11 studies) was 1.51 compared with those without pre-transplant cancer (95% CI 1.28–1.80; I^2^ = 74%), 3.13 (95% CI 2.29–4.27; I^2^ = 71%) for cancer-specific mortality (3 studies) and 4.64 (95% CI 2.54–8.49; I^2^ = 74%) for the development of *de novo* post-transplant cancer (6 studies) [25]. An association between pre-transplant cancer history and increased risk of all-cause cancer and non-cancer-specific mortality was also observed in subsequent studies [24, 26, 27]. Challenging the accepted paradigm of recommending a pre-specified waiting time for patients with prior cancer before transplantation, a Norwegian study showed that recipients with a pre-transplant cancer (with a required waiting period of 12 months) had similar allograft and patient survivals compared with recipients without prior cancers [28]. While the authors did not find an association between waiting time and all-cause and cancer-specific mortality, cancer-related mortality was at least 3.5 times higher in the first 5 years of follow-up (HR 3.44; 95% CI 2.36–5.03) compared with patients without cancer.

As the field of transplantation has evolved momentously in the last decade, particularly in the era of precision oncology, setting a rigid and inflexible cancer-free waiting time and using a ‘one-size-fits-all approach’ for all transplant candidates with prior cancer history is no longer acceptable. A simplistic and practical approach to define transplant suitability is to estimate the likelihood of ‘cancer cure’ within a pre-determined period of observation of cancer remission. For example, for patients with localized cancer and treated with curative approaches, we suggest a waiting period of no more than 2 years; for those with regionally spread cancer and who are cured from the disease, a waiting period of no more than 5 years may be considered. For patients with metastatic cancers and who are in remission for more than 5 years after curative treatment, transplantation may be contemplated if there is no evidence of disease recurrence. However, other factors such as the types, grade, stage and the molecular features of the cancer should also be included in the decision-making process. While an acceptable cancer-free interval prior to transplantation is difficult to define, the recurrence-free period should exceed the time deemed the highest risk of cancer recurrence. In addition, transplant eligibility decisions must balance the ultimate desire to provide potentially life-changing procedures to those with kidney failure against the potential harms associated with cancer recurrence and death. In addition, involvement of a multidisciplinary team such as allied health professionals, clinical nurse specialists, oncologists, nephrologists and surgeons are crucial to enhance the continuing care pathways before and after transplantation, ensuring appropriate surveillance procedures are available for those at risk of recurrence.

Nevertheless, there are several issues that must be carefully considered in the discussion of transplant suitability for patients with a prior cancer history.

### Defining the risk of cancer recurrence and cancer-related mortality (following cancer recurrence) in the era of novel anti-cancer therapies

**Figure 1:**
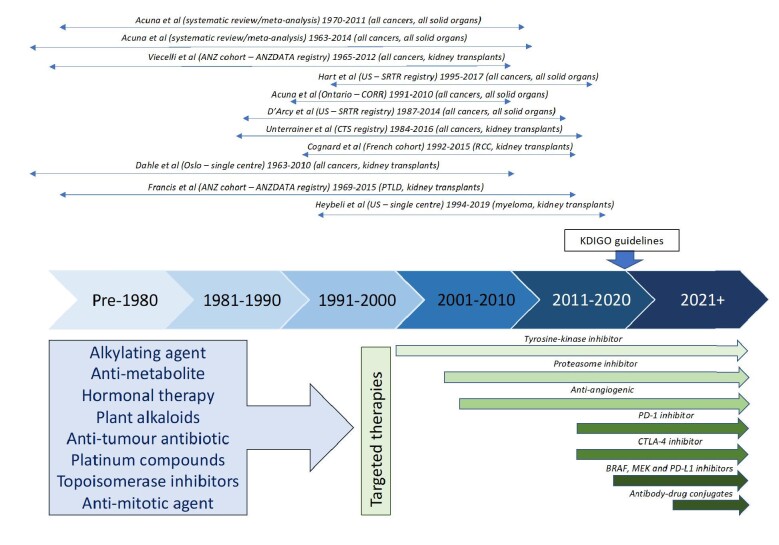
Timeline of novel anti-cancer drug approval and cohort studies of kidney transplant recipients with prior cancer history.

While chemotherapy and radiotherapy are considered the most effective and widely used modalities in treating cancers, the rapid and dynamic development of many novel anti-cancer therapeutics, such as targeted drug therapy, gene therapy, cancer stem cells therapy, immunotherapy and ultrasound-mediated drug therapy to deliver cancer therapeutics, has revolutionized cancer management in patients with advanced stage disease. Figure 1 shows the timeline of anti-cancer drug approval over time. For example, metastatic breast cancer is considered as uncurable, but with the availability of cancer immunotherapy (immune checkpoint inhibitors such as anti-CTLA4, anti-PD-1 and anti-PD-L antibodies) and antibody drug conjugates, patients with metastatic breast cancer may achieve remission. Case series have reported a 5-year survival of approximately 30%–50% in women with liver metastases treated with a combination of ipilimumab and anti-PD-1 antibodies [29]. The current guidelines suggest patients with a history of metastatic disease should be excluded from transplant listing, but with the advent of these novel therapies, complete response and remission can be achieved. The question of re-listing and re-transplantation should therefore be a shared decision-making process between the transplant health professionals, patients and their families, taking into consideration the risk of disease recurrence and death after disease recurrence in the context of re-transplantation and immunosuppression use, patients’ values and views, preferences [30], and their overall quality of life and survival treated with maintenance dialysis. Given that cancer recurrences are a rare occurrence, concerted effort by the global transplant community to collect important patient outcome data including survival, quality of life, allograft function and any adverse effects will inform treatment decisions.

### Defining the optimal and frequency of cancer surveillance in patients with prior cancers to exclude occult cancer recurrence

One of the key goals of routine surveillance after cancer treatment cure is to detect and identify asymptomatic relapse or recurrence of diseases such that therapeutic and curative strategies can be implemented early and effectively in order to improve clinical outcomes such as survival. These surveillance approaches may include imaging, blood test, bio- and tumour markers assessments, and other invasive tests such as colonoscopies, which may incur costs and potential harms. In addition, undertaking these routine surveillance strategies may be burdensome to patients. Therefore, an individualized, risk-based surveillance strategy that targets patients at risk of having disease recurrence will guide the frequency and the modality of testings in these individuals. Several reliable prognostic genetic algorithms have been developed to identify patients at risk of disease recurrence and to guide treatment and surveillance strategies. For example, the Oncotype DX 21-gene recurrence score is known to be a sound prognostic and predictive assay in node-negative hormonal receptor-positive and human epidermal growth factor receptor 2 (HER2)-negative breast cancer patients [31].

### Avoidance of high immunological risk donor kidney and avoidance of T-cell-depleting agents as induction therapy

Prior research has indicated that transplant recipients who experienced acute rejection and received T-cell-depleting therapies experienced a 1.4-fold increased risk of developing cancer, with an excess risk of approximately 2.2 times for genitourinary tract cancers compared with recipients with no prior acute rejection episodes [32]. T cells are known to have a vital role in adaptive immunity and therefore depletion of T cells is likely to lead to a greater risk of infections and cancers. More importantly, CD4+ T helper 1 cells, CD8+ cytotoxic T cells and CD16+ natural killer cells, are the key antitumour effector cells [33]. Therefore, in potential candidates with a prior history of cancer, better immunological matched donor kidneys (deceased and living kidney donation), and other induction therapies such as the use of interleukin-2 receptor-alpha blockers with basiliximab, may be preferred to reduce the overall risk of acute rejection.

### Consideration of the risk of non-caner related death on dialysis and balance against the risk of cancer recurrence after transplantation

Patients treated with maintenance dialysis are at risk of death from cardiovascular-related disease (CVD). The relative risk of death from CVD is at least 20 times higher than that in the general population. Many of our patients succumb to cardiac-related events and death while waiting for a kidney transplant [34]. Transplantation, on the contrary, may reverse these vascular risks, improve overall quality of life and post-transplant survival. In patients with prior cancer, the survival gains may be limited by the potential risk of cancer recurrence. Therefore, it is important to quantify the risks and the anticipated benefits about transplantation and inform our patients the alternatives (remain on dialysis) so they can make informed decisions about their own treatment choices.

## CONCLUSIONS

Cancer is a debilitating disease in transplantation recipients because it is the second leading cause of death. Patients with a prior cancer history have an increased risk of developing cancer after transplantation and a higher risk of cancer-related death compared with recipients without a previous cancer. While complete cancer remission is an essential criterion for transplantation, the risk of disease recurrence is dependent on the cancer types, sites and stage of the disease at first diagnosis. Cancer management is particularly challenging for transplant health professionals and patients with kidney transplants. Apart from the intricacy of cancer care, clinicians and patients often have conflicting priorities of achieving cancer cure, maintenance of allograft function and prevention of allograft loss. A shared decision-making process that allows transplant recipients with cancers to better understand the management options, the potential trade-offs between over and under-immunosuppression, and the risks and benefits of cancer treatments (such as acute rejection and acute kidney injury) are essential because this will ensure that patients’ values and preferences are aligned and incorporated into complex treatment decisions.

## Data Availability

No new data were generated or analysed in support of this research.
